# The importance of brain mapping for rehabilitation in birth nonprogressive neuromuscular diseases

**DOI:** 10.3389/fnimg.2024.1359491

**Published:** 2024-07-15

**Authors:** Aleksandra Tolmacheva, Olga Agranovich, Evgeny Blagovechtchenski

**Affiliations:** ^1^Center for Cognition and Decision Making, National Research University Higher School of Economics, Moscow, Russia; ^2^G.I. Turner Scientific Research Institute for Children's Orthopaedics, Ministry of Health of Russia, Saint Petersburg, Russia

**Keywords:** motor mapping, birth motor disorders, transcranial magnetic stimulation, rehabilitation, neuromodulation, cortical plasticity

## Abstract

While motor mapping has been extensively studied in acquired motor conditions, a lack has been observed in terms of research on neurological disorders present since birth, with damage to the spinal cord and peripheral nerves (hence, defined in this study as nonprogressive neuromuscular diseases). Despite an injury at the level below the brain, the subsequent changes in the motor system involve cortical reorganization. In the scientific community, the need for a comprehensive approach targeting the brain is increasingly recognized for greater motor recovery in these patients. Transcranial magnetic stimulation (TMS) and functional magnetic resonance imaging (fMRI) are the most utilized techniques for motor mapping. The knowledge obtained through motor mapping may be used to develop effective individual neuromodulation therapy that helps in functional motor recovery. This brief review compares the results of the brain mapping of a few existing studies in individuals with nonprogressive motor disorders of nonbrain origin present at birth to the brain mapping of individuals with similar acquired motor conditions. The review reveals some particular features in terms of central adaptation in individuals with birth conditions compared to their acquired counterparts, such as the nonsomatotopic presentation of involved muscles in the sensorimotor cortex and nonadjacent cortical areas. This topic is undoubtedly intriguing, justifying further research in the field. This review also discusses the benefits these patients can obtain from neuromodulation therapy addressed to the central nervous system and the importance of individual neurophysiological assessment in designing rehabilitation therapy for children with birth motor disorders.

## 1 Introduction

Motor deficit (referred to as birth deficit/disorder in this text) observed in newborns can be caused by congenital disease or obstetrical trauma. A congenital disease is a disorder that occurs during intrauterine life with underlying genetic factors, chemical or physical intrauterine exposure, or infections during pregnancy. A birth injury results from excessive mechanical force during delivery. Although these conditions differ in terms of etiology, their common aspect is a child's impaired postnatal motor development from birth. In this review, studies on birth motor deficits—both congenital and obstetrical etiology—were sought. Such disorders are referred to as birth nonprogressive neuromuscular diseases, and they are associated with damage to the level of the spinal cord and the peripheral nervous system. Examples of such disorders include indicative diseases, such as arthrogryposis multiplex congenita (AMC) and obstetrical brachial plexus palsy (OBPP). AMC, antenatal neuropathies and injuries to the spinal cord are congenital neurological motor conditions. The incidence rate of AMC is reported to be 0.03–0.1 per 1,000 live births, and the statistics of nonprogressive antenatal neuropathies and spinal cord injuries are difficult to express from the entire group of congenital spinal cord injuries and neuromuscular diseases with a progressive course. For birth traumatic motor deficits, OBPP is a common pathology, with an incidence rate of 0.4–4 per 1,000 live births (O'Berry et al., 2017). In severe cases, birth defects significantly reduce limb functionality, leaving a child disabled, which is particularly evident in the marked changes in the body of a growing child. In such cases, surgical interventions for adjusting bones, joints, and muscles, as well as muscle and nerve transfer, are performed already. Preferably, intensive postoperative rehabilitation should follow surgery to establish functional neuronal circuits underlying newly acquired motor skills (Simon et al., 2015). Conventional rehabilitation, such as physical therapy, can be empowered with stimulation approaches (Evancho et al., 2023), which is of particular relevance for children of early age, as the surgical treatment of birth conditions starts in early childhood. At an early age, children are unlikely to benefit from physical therapy alone, as they are incapable of performing assigned active motor tasks. In such cases, neuromodulation techniques can be successfully used to realize the full potential of the performed surgery (Socolovsky et al., 2017). Since the origin of these diseases are located at the level of the peripheral nervous system and the spinal cord, the latter are the main targets for treatment. Nonetheless, this review proposes that higher levels of the nervous system should also be considered important for targeted treatment. Thus, acquaintance with plastic processes in these patient populations is essential for developing effective neuromodulation therapy. Motor mapping is the method through which motor function is localized in the brain cortex. As an output, the method of motor mapping generates comprehensive brain maps that represent the specific neuronal networks involved in particular motor activities. For instance, neuroimaging techniques such as functional magnetic resonance imaging (fMRI), functional positron emission tomography (fPET), magnetoencephalography (MEG), electroencephalography (EEG), and single-photon emission tomography (SPECT) allow for visualization throughout the brain during the imagining or performance of motor tasks (Shibasaki, 2008). These features are particularly interesting since they allow for a holistic approach. In fact, active voluntary movements engage brain activity at the network level (Nazarova and Blagovechtchenski, 2015). However, the application of these techniques for targeted neuromodulation therapy is limited due to their insufficient spatiotemporal resolution that restricts the obtained information on the cortical localization of particular muscles (Rossini et al., 2007). In contrast, transcranial magnetic stimulation (TMS) is a brain stimulation method that can artificially activate the corticospinal tracts (CSTs), providing passive brain mapping when the muscles are at rest. Among other methods that allow for motor mapping, TMS exclusively exhibits high spatiotemporal resolution, allowing for the dissection of separate muscle representations in the cortex (Picht, 2015). Therefore, TMS seems to be a method of choice when selective brain targets must be non-invasively defined for stimulation therapy and diagnoses during motor rehabilitation (Rossini et al., 2007). TMS is a more affordable technique than neuroimaging methods, which also demonstrates safety and feasibility in children population (Krishnan et al., 2015).

A large number of motor mapping studies have been performed on healthy individuals and those with motor conditions (acquired motor disorders, congenital disorders involving the brain) (Lefaucheur, 2019; Zelenski et al., 2023), while some motor diseases remain unexplored. The goal of this work was to review research on motor mapping in birth nonprogressive motor disorders with non-brain origins, meaning its implementation in designing targeted neuromodulation therapy. This review has not included congenital progressive neurological diseases, as their cortical adaptation presumably proceeds distinctly due to continuous pathophysiological changes over the course of the disease. This can be a topic for another review. In this review, studies on motor mapping that utilized the aforementioned neuroimaging methods and TMS were searched. This review discusses how the knowledge of cortical plasticity obtained through brain mapping is important for personalizing neuromodulation therapy to achieve functional motor recovery in these patients.

## 2 Motor mapping of individuals with birth nonprogressive neuromuscular diseases

Injury to the motor system at any level causes immediate reorganization of the involved motor tracts (Simon et al., 2016; Needle et al., 2017; Mohammed and Hollis, 2018). Typically, such tracts functionally connect different levels of the central nervous system, from the cerebral cortex to the motor neuron. Injury-related plasticity depends on the nature and severity of the injury and the patient's age. In general, neuroplasticity increases the motor threshold, shrinkage of the cortical muscle representations of the affected muscles, and expansion of the cortical muscle representations of intact muscles (Zdunczyk et al., 2018). However, maladaptive neuroplasticity may primarily be observed in very young children due to highly intensive plastic processes. For instance, muscle cocontraction in OBPP may develop due to aberrant reinnervation and the subsequent activation of neighboring cortical areas (Zelenski et al., 2023). Considering the highly possible formation of maladaptive neuroplasticity in children, it is imperative to conduct neurofunctional studies, including motor mapping, to develop an effective rehabilitation program in the pediatric population.

The cortical level plays a critical role in organizing and controlling precise movements. Cortical somatotopy arrangement is driven conjointly by genetic programs with individual motor experience (Forssberg, 1999). Accordingly, individuals born with motor disabilities are expected to exhibit different features in the sensorimotor cortex (SM) organization than those with acquired motor disorders. For instance, it can be observed when comparing motor disorders at birth with their acquired counterparts. Of congenital motor disabilities, cerebral palsy (CP) is profoundly studied (Wittenberg, 2009) in which a lesion to the brain occurs antenatally or perinatally. When CP occurs due to a perinatal stroke, the neurological impairment resembles that caused by a postnatal stroke, still holding some different plastic features. As such, perinatal stroke patients exhibit more prominent ipsilateral corticospinal projections to the less affected side than postnatal stroke patients (Nardone et al., 2021). In addition, children with unilateral motor deficits exhibit activation in ipsilateral corticospinal projections (Zuniga et al., 2021). Hence, it is worthwhile to collect MEPs bilaterally to investigate the potential of unilateral pathways, as treating the unaffected hemisphere can promote rehabilitation of the injured side through cross-education (Ruddy and Carson, 2013).

Unlike lesions at the level of the brain cortex in perinatal stroke patients, birth motor disorders caused by lesions to the spinal cord and peripheral nerves appear with secondary cortex reorganization (Raineteau and Schwab, 2001; Navarro, 2009). OBPP is caused due to injury to the brachial plexus during delivery, which, in severe cases, leads to permanent disability. Evidence of a change in the upstream sensorimotor tract has been found, with eventual cortical reorganization following a traumatic brachial plexus injury, which has been demonstrated in several fMRI studies on dynamic cortical rearrangement over recovery. Cortical activation was observed predominantly within the SM contralaterally to the damage (Khu, 2015). To the best of our knowledge, no TMS studies have been conducted on the brain mapping of such patients. One study was performed on adult individuals with a residual motor deficit in shoulder function due to OBPP, in which cortical activation was investigated with fMRI during motor performance. The study found bilateral activation in the primary motor cortex (M1) and the associated sensorimotor areas when the injured hand was moving (Björkman et al., 2016). Notably, cerebral activation in SM during motor tasks expanded posteriorly into the parietal lobe in OBPP individuals compared to those who had traumatic brachial plexus injury (Yoshikawa et al., 2012; Li et al., 2015).

Upper limb reduction defects (ULRDs) constitute a congenital peripheral sensorimotor deficit that, to some extent, is also present in OBPP. It is assumed that secondary cortex reorganization in ULRD may serve as a bright illustration of dramatic sensorimotor deficits that can provide an approximation of the cortical plasticity profile in OBPP. Several reports exist on the motor mapping of patients with ULRDs. One fMRI study made a direct comparison of hand representations between handless individuals with acquired and those with congenital conditions (Wesselink et al., 2019). In the study, the extent of the motor and sensory hand representation areas and the level of activation in the SM were lesser in individuals with congenital hand absence than in those with hand amputees. In addition, another fMRI study showed differences in SM organization between hand amputees and ULRD, suggesting that cortical plasticity is restricted by developmental stage (Root et al., 2022). Furthermore, a Stoeckel et al. (2009) fMRI and TMS study demonstrated a non-somatotopic foot cortical scheme in patients with ULRD. The adult subjects exhibited fine-skilled foot functionality that compensated for non-functional hands. Brain mapping revealed—along with the typical location of the foot muscle in the medial precentral gyrus—non-somatotopic representation of the foot muscle in the non-adjacent cortex, laterally in M1, overlapping with the hand area. Furthermore, an interesting report was made in a case study investigating a woman with amelia in the upper and lower extremities (Brugger et al., 2000). The woman, born without legs and forearms, experienced vivid sensory and movement phantoms. Similar to the Stoeckel study, activation during phantom hand movement was found in non-somatotopic areas (i.e., the premotor and parietal areas), although there was no activation in M1. Activation of the existing upper arms showed expansion through the hand area in M1. These two studies on congenital limb defects demonstrated the tremendous capacity of the nervous system for plastic adaptation, which is unlikely to be seen in amputees and highlighted the difference between motor maps in congenital and acquired conditions.

Hence, no studies on motor mapping employing neuroimaging techniques or/and TMS in patients with other nonprogressive developmental anomalies of the spinal cord and peripheral nerves were found.

## 3 Discussion

One interesting study on ULRD demonstrated a direct comparison of birth and acquired hand absences (Wesselink et al., 2019). This study highlighted a dissimilarity in the *similar* clinical presentation of birth and acquired motor conditions and the importance of individual neurophysiological assessments of patients with birth conditions. This brings us back to the neuroscience principle of forming functional systems based on inherited anatomical substrates conjointly with individual experience. Because the nervous system of humans is naturally immature at birth and for many years before it reaches adulthood, deafferentation and deefferentation accompanying postnatal development impact the formation of motor programs. Few studies have explored motor behavior in individuals with OBPP (Brown et al., 2000; Anguelova et al., 2016). In the study by Anguelova et al., disrupted spontaneous motor reactions were found in provocation tests in which subjects were capable of executing motor tasks only with attention. The researchers proposed that this might be related to the impaired development of central motor programs. This highlights that, in patients with motor disorders, physical competence alone is insufficient to perform movements normally. Instead, these patients need to relearn deviant motor skills for optimal movements, and this relearning is implemented via central nervous plasticity despite the peripheral origin of the disease.

To achieve the full potential of rehabilitation, surgical treatment should be consolidated jointly with postsurgery motor rehabilitation aimed at training new motor skills. Hence, in the clinical community, neuroplasticity is becoming a recognizable concept underpinning favorable outcomes in motor rehabilitation, as supported by neuroimaging studies on nerve transfer in patients with brachial plexus injury, which highlighted the importance of brain plasticity, notwithstanding the peripheral origin of the disease (Socolovsky et al., 2017). Modern motor rehabilitation comprises physical therapy accompanied by neuromodulation approaches (Wendt et al., 2022). The latter requires an understanding of neurophysiology to develop effective protocols. Neuromodulation therapy employs this understanding to qualify the personalized protocol with stimulations of a certain mode (facilitating or inhibiting) delivered at a precise site in the cortex to fulfill rehabilitation needs (Gao et al., 2024). On the one hand, such customized protocols can manage maladaptive neuroplasticity and, on the other hand, govern the formation of new, desirable neuronal pathways after surgical treatment. Resultant neuroplasticity can not only be limited to the CST but also induced at the cortical level. For instance, an examination of intracortical excitability performed with repetitive TMS may indicate abnormal excitation in neuronal circuits. Furthermore, stimulation protocols can be applied to balance the detected abnormal neuronal activity (Mann and Malhi, 2023). Brain mapping also allows for the detection of plastic changes occurring upon intervention, resulting in motor map dynamics that can mark the effectiveness of a given therapy.

Because birth motor deficits impact the normal development of motor programs, it is critical to begin therapeutic intervention early in childhood when plastic processes are the most responsive. Thus, a comprehensive approach targeting multiple sites (e.g., the brain cortex, the spinal cord, and the musculoskeletal apparatus) seems to be the most constructive way to interfere with deviant motor progression. Moreover, earlier therapy prevents secondary bone and joint deformation, significantly impacting hand functionality.

Furthermore, motor mapping can facilitate the selection of the best muscle candidate for muscle transfer during a presurgical evaluation. Two factors for successful muscle transfer have been discussed in the literature (Yoshikawa et al., 2012). First, it is hypothesized that a greater cortical representation of the donor muscle would readily contribute to the formation of new corticospinal pathways for the affected muscle. Second, the closer the cortical layout of the donor and recipient muscle representations, the higher the probability of establishing a successful connection between the two cortical areas through which the affected muscle would gain functionality.

## 4 Conclusion

It has emerged that there is scarce research on brain mapping, which does not permit drawing a clear conclusion on neuroplastic dynamics in children with birth motor deficits of non-brain origin. Considering that this review revealed a lack of research performed on these patients, brain stimulation therapy is unlikely to be applied to this patient group, despite performing surgical treatment. Evidence from several reports shows that there are particular quantities in central adaptation in children with birth motor deficits compared to those with similar acquired motor conditions. In addition, the evolving deviant central motor programming in children with birth motor deficits, which contributes to consequent developmental motor disorders, is discussed in the literature. Overall, a demand seems to exist for a specific approach to rehabilitation in the pediatric population and the importance of the neurofunctional assessment of each patient being considered for therapy.

In contrast, functional brain motor mapping has been extensively studied in numerous acquired motor disorders. This review opposed birth and acquired conditions to apprise clinicians of different central adaptations in these two groups of patients. Thus, the review aims to secure the rehabilitation team against interpolation of the existing knowledge to the group of birth disorders when designing neuromodulation therapy.

## Author contributions

AT: Conceptualization, Writing – original draft, Writing – review & editing. OA: Writing – review & editing. EB: Conceptualization, Writing – review & editing.
